# Neurodevelopmental Impact of Prenatal Stress: A Proteomic Analysis of Myelination Disruptions in the Avian Embryo

**DOI:** 10.1002/dneu.23003

**Published:** 2025-10-14

**Authors:** Bela Gaertner, Gabriela Morosan‐Puopolo, Beate Brand‐Saberi, Charmaine Schücke, Darius Saberi, Katharina Klöster, Simon Faissner, Katrin Marcus, Morris Gellisch, Britta Eggers

**Affiliations:** ^1^ Medizinisches Proteom‐Center, Medical Faculty Ruhr University Bochum Bochum Germany; ^2^ Medical Proteome Analysis, Center for Protein Diagnostics (PRODI) Ruhr University Bochum Bochum Germany; ^3^ Department of Anatomy and Molecular Embryology, Institute of Anatomy, Medical Faculty Ruhr University Bochum Bochum Germany; ^4^ Department of Neurology Ruhr University Bochum, St. Josef‐Hospital Bochum Germany; ^5^ Department of Neurology University Medical Center, Georg August University Göttingen Germany; ^6^ Fraunhofer Institute for Translational Medicine and Pharmacology Göttingen Germany; ^7^ Center for Medical Education Ruhr University Bochum Bochum Germany

**Keywords:** corticosterone | *Gallus gallus domesticus |* myelin | neurodevelopment | prenatal stress | proteomics

## Abstract

Prenatal stress, mediated by elevated glucocorticoid (GC) levels, is a relevant modulator of fetal brain development and a known risk factor for neurodevelopmental disorders. Using the avian embryo as a vertebrate model, we injected corticosterone into the yolk on embryonic day 6 (E6) and assessed neurodevelopmental outcomes at day 14 (E14). Through deep proteomic profiling — quantifying over 6500 proteins — we uncovered a robust molecular signature of stress‐induced disruption. Key myelin‐associated proteins (myelin basic protein [MBP], PLP1, 2′,3′‐cyclic‐nucleotide 3′‐phosphodiesterase [CNP]) were markedly downregulated, indicating impaired oligodendrocyte maturation. These proteomic shifts were corroborated by immunohistochemistry and qPCR. Pathway‐level analysis pointed to altered MAPK and AKT signaling as putative mediators of the observed phenotype. Our findings mirror previous mammalian data while highlighting the avian model's unique suitability for mechanistic dissection of prenatal stress effects. This study offers new insight into how early GC exposure impairs glial development, with broader implications for understanding the molecular origins of stress‐linked brain vulnerability.

## 1 Introduction

Prenatal stress refers to the disruption of homeostasis in the maternal organism, which, in turn, affects the fetus in utero. When the maternal organism experiences this disbalance, it can trigger the activation of the hypothalamic–pituitary–adrenal (HPA) axis, leading to the secretion of glucocorticoids (GCs) into the maternal circulatory system. These GCs, which are steroid hormones, can — within limited amounts— cross the placental barrier and influence the fetus during critical periods of its development (Gitau et al. 2005; Zhu et al. 2019). GCs play a crucial role in regulating a range of vital physiological processes, including energy metabolism, immune function, and cardiovascular regulation (Bosscher and Haegeman 2009; Krugers et al. 2012; Nussinovitch et al. 2010; Vegiopoulos and Herzig 2007). Moreover, higher cognitive brain functions, for example, mood, memory, and cognition, can be affected (Farrell and O'Keane 2016; Joëls 2011).

The deleterious effects of prenatal stress in humans and other mammals can range from low birth weights to increased risk of behavioral and cognitive problems later in life, such as depression, anxiety, and ADHD (Arcego et al. 2024; Broberg et al. 2018; Koenig et al. 2002; Lautarescu et al. 2020; Xu et al. 2013). Recent studies have revealed that external factors inducing stress, such as loud noises, lack of food, and extreme heat or cold, can not only result in an increase in maternal GC stress hormones but also alter the fetus's HPA axis responsivity (Gui et al. 2024; Jafari et al. 2017; Lindsay et al. 2017). Furthermore, during pregnancy, GC levels may rise drastically, thus making pregnancy itself “hypercorticolistic”. In humans, maternal plasma cortisol levels may also increase two‐ to threefold. As a barrier against these increased levels of stress hormones, the enzyme 11β‐hydroxysteroid‐dehydrogenase (11β‐HSD), which catalyzes the reaction of active steroids to inert steroids, is expressed in the placenta of humans as well as in other mammals. Despite this protective barrier, there is evidence that around 10%–20% of maternal GC still passes through to the developing fetus, where high concentrations, due to stress‐induced elevated maternal GC, may be sufficient to result in various short‐ as well as long‐term effects (Jahnke et al. 2021). Furthermore, studies on placental cells have shown that the elevation of cytokines due to infections suppresses the expression of 11β‐HSD, which, in turn, can lead to significantly increased concentrations of GC (Kossintseva et al. 2006). Behavioral studies have convincingly shown that maternal stress during pregnancy leads to both short‐ and long‐term adverse effects on offspring, particularly in neurological development (Jagtap et al. 2023). This suggests that prenatal stress can significantly impact the development of the offspring's brain (Weinstock 2008). Confirmatively, morphological studies in mammalian model systems observed ultrastructural changes in the hippocampus, including irregularly shaped nuclei, vacuole formation from swollen and distorted mitochondria, and chromatin condensation in stressed rat pups (Xu et al. 2013). Furthermore, alterations in hippocampal myelination could be verified in rat as well as in guinea pig models (Crombie et al. 2023; Xu et al. 2013). Thus, studies focusing on elucidating the molecular mechanisms of controlled stress exposure on the developing brain are of utmost importance. However, in mammals, the maternal organism influences the GC levels of the fetus, making it extremely challenging to administer GC in a controlled manner for studying their effects on fetal development. The fact that husbandry conditions of mammalian research organisms, mainly rats, often already impose a great amount of stress on these animals, and the relative inaccessibility of the fetuses, further complicates research on prenatal stress in mammals. Therefore, the use of non‐mammalian vertebrates, such as birds, where development occurs without contact to the maternal organism, presents promising advantages for future investigation of prenatal stress.

One of the most prominent higher vertebrate models is *Gallus gallus domesticus*. The embryos of *G. gallus domesticus* have long been used in fields such as immunology, embryology, and cell biology, and are in fact one of the oldest animal models, having a rich history of over 2000 years. In 2004, the chicken genome became the first non‐mammalian amniote genome to be sequenced, filling a much‐needed gap between the sequenced mammalian genomes and other vertebrate genomes. Intriguingly, the chicken genome (∼1 billion bp) is much more compact when compared to the human genome (∼2.8 billion bp). However, it seems to encode for a similar number of genes (roughly 23,000). Approximately 60% of its genes correspond to a human homolog, having a sequence identity of 75% on average. Additionally, epigenetic marks, such as histone modifications and DNA methylation, share similar patterns in chicken and human (Beacon and Davie 2021). The embryonic development of *G. gallus* *domesticus* takes approximately 20 to 21 days after the egg is laid and begins at its conception (Hamburger and Hamilton 1992). Critical points of development must be considered, depending on the research question. In the present study, we applied a single corticosterone injection at embryonic day 6 (E6) to model an acute prenatal stressor. E6 marks a critical window in brain development, as major brain structures are initiated, but the macroscopic differentiation of the cerebellum, cerebral hemisphere, interhemispheric fissure, optic lobe, pineal body, and the transverse fissure is still underway (Selcuk and Kayikci 2025).

During the rest of the development, in our case until day 14 (E14) of development, the different brain regions continue to develop in size and organization. In a pioneering study on embryonic development, we demonstrated that glucocorticoid receptor (GR) expression emerges as early as day 2 (Hamburger–Hamilton stage 13, HH13) in the chicken embryo (Bablok et al. 2023). Further, GR expression during neurulation in the neural folds and the neural tube indicated a role of GC signaling during neural development (Bablok et al. 2023). Confirmatively, GR expression was verified in the prosencephalon, mesencephalon, and the diencephalon (Bablok et al. 2023), laying the basis that the chicken embryo represents a good model organism to study prenatal stress in early developmental stages. In a more detailed investigation, the morphological analysis of embryos exposed to prenatal stress through corticosterone revealed significant developmental defects, including reduced overall embryo size, ectopic heart formation, and abnormalities in skin morphogenesis, clearly demonstrating the severe impact of stress on embryonic development (Bablok et al. 2022; Gellisch et al. 2023).

Although *G. gallus domesticus* represents one of the major model organisms in the field of developmental biology, few studies have investigated its proteomic composition. This could be due to the fact that until recently, there was only one species with a sequenced genome (Ferver et al. 2022), complicating the proteomic characterization. Since then, an increasing number of avian genomes have been sequenced, allowing the proteomic study of the avian proteome. To date, most studies on the avian proteome have focused on characterizing the proteomic composition of egg white, yolk, shell (Liu et al. 2021; Liu et al. 2020; Mann and Mann 2011; Saadeldin et al. 2023; Shi et al. 2023), and cerebrospinal fluid (CSF) (Bueno et al. 2020; Voukali et al. 2021). Furthermore, the food industry shows great interest in proteomic studies to investigate factors contributing to improving feed conversion efficiency, food quality, and the health and welfare of animals. Further, several studies focused on the elucidation of the proteomic composition of defined organs, such as gonads (Qin et al. 2022) and liver (Yang et al. 2021), as well as skeletal muscle tissue (Ouyang et al. 2017). Few studies have explored the proteome of the developing chicken brain; however, one pioneering study focused on characterizing the midbrain, identifying 5929 proteins following sample fractionation (Stepler et al. 2023). In our study, we have expanded the identification of proteins in the developing chicken brain, quantifying 6581 proteins from unfractionated samples, making this one of the most extensive proteomic analyses conducted on the developing chicken brain. Using protein‐based mass spectrometry, we identified molecular alterations resulting from corticosterone exposure, with analysis revealing deficits in myelination as a prominent pathological hallmark, consistent with findings from previous mammalian studies.

## 2 Materials and Methods

### 2.1 Chicken Embryo Treatment

The treatment of chicken embryos is described in detail in Gellisch et al. (2023). In brief, fertilized chicken eggs were purchased from a local breeder, disinfected, and incubated (37°C, 80% relative humidity). A total of 50 fertilized chicken eggs were divided into a corticosterone‐exposed group (CORT, *n* = 25) and a control group (CTRL, *n* = 25). On the third day of embryonic development (E3) (Stages HH20–HH21), 3 mL of albumen was removed with a syringe. On E6 of incubation (Stages HH28–HH30), 15 µg of corticosterone (Sigma‐Aldrich, St. Louis, MO, USA) dissolved in 100 µL phosphate‐buffered saline (PBS) with 1% ethanol was injected into the yolk sac, leading to a systemic administration of the GC. For the experimental control, 100 µL PBS with 1% ethanol was injected. The corticosterone dose used in this respective research design was based on previous investigations (Heiblum et al. 2001) and additional dose‐ranging experiments aimed at mimicking pathologically elevated GC concentrations. On day 14 of incubation, the embryos were isolated from the eggs. Brains were surgically removed and either microscopically imaged for morphological analysis (CTRL *n* = 8, CORT *n* = 4) or flash frozen in isopentane precooled in liquid nitrogen for further proteomic analysis (CTRL *n* = 6, CORT *n* = 5). Embryo survival rates were monitored throughout the incubation period. Although natural batch‐to‐batch variations occasionally affected control groups due to environmental factors, corticosterone treatment consistently resulted in approximately a 20% higher mortality rate compared to controls.

### 2.2 Ethical Statement

According to German law, animal experimentation approval is only necessary for embryonic vertebrates when they have reached the last third of their development. In the case of chickens, any experiments performed before embryonic day 14 (E14) are not classified as animal experiments under the Tierschutzgesetz and therefore do not require formal approval or government authorization.

### 2.3 Morphological Analyses and Immunohistochemistry (IHC)

IHC was performed to detect the GR in embryonic tissue sections. Tissue sections (8 µm) were deparaffinized using a graded series of xylol and ethanol washes, followed by antigen retrieval in a citrate buffer (19 mM, pH 6.0) in a microwave for 6–8 min at 1000 W until boiling, then maintained at 300 W for 15 min. After cooling, sections were blocked with 3% BSA in PBS for 1 h at room temperature. The primary antibody, Glucocorticoid Receptor Antibody (MA1‐510, BuGR2, Product # MA1‐510, Thermo Fisher), was applied in a 1:30 dilution overnight at 4°C in a humidified chamber. Following primary antibody incubation, sections were washed and incubated with an Alexa Fluor Plus 488‐conjugated secondary antibody in a 1:1000 dilution (Mouse IgG, H+L, Highly Cross‐Adsorbed, Product # A32723, Thermo Fisher) for 1 h at room temperature in the dark. After further PBS washes, the sections were mounted with Roti‐Mount FluorCare DAPI and stored in the dark at 4°C until imaging. Fluorescent images were captured using a fluorescence microscope (Zeiss Axio Scan Z1), with GR expression visualized as green fluorescence and blue‐stained nuclei (DAPI). The sections were microscopically analyzed and imaged using the virtual slide microscope VS120 (Olympus, Tokyo, Japan). To assess region‐specific differences in GR expression, a semiquantitative analysis was performed on IHC images of midbrain sections. Images were converted to 8‐bit grayscale, and the green fluorescence channel corresponding to GR immunoreactivity was analyzed using ImageJ v1.53 (Schneider et al. 2012). Five rectangular regions of interest (ROIs) of equal size (2500 px^2^) were placed in anatomically comparable locations within the midbrain for each sample in both control and stress‐exposed groups. Mean gray values were extracted from each ROI, and for each individual, the average value across the five ROIs was calculated. Group differences were assessed using an unpaired two‐tailed Student's *t*‐test. A *p*‐value <0.05 was considered statistically significant.

Staining of myelin basic protein was carried out (Product # ab2180111, abcam, dilution 1:500). For this purpose flash frozen tissue was cryosected (15 µm), fixed in 100% aceton for 30 min,‐ and incubated with the primary antibody overnight. Secondary antibody incubation (AlexaFluor 532 Rabbit, Product # A11009, Invitrogen, dilution 1:200) was carried out after three intensive wash steps with PBS for 1 h at RT. The Olympus VS120 BX61VS virtual slide microscope and OlyVIA software (version 2.9) were used for fluorescence imaging.

A two‐dimensional brain area determination was carried out in ImageJ with four brains from the CORT group and eight brains from the CTRL group using light microscopy pictures taken from the dorsal side. Measurement of brain areas was realized utilizing images of whole brains. The two telencephalic lobes were measured as a single unit, whereas optic lobe measurements were averaged due to the anatomical distance between them. In addition, the total dorsal brain area and the cerebellum were measured separately. Statistical significance was determined using Welch's *t*‐tests. A *p‐*value <0.05 was considered statistically significant.

### 2.4 Protein Lysis and Digestion

Frozen brains (*n* = 11) were lysed as previously described (Eggers et al. 2021). In brief, frozen brains were weighed and homogenized in liquid nitrogen using a mortar. Homogenates of 100 mg were transferred to a new reaction tube and lysed with DIGE buffer (7 M urea, 2 M thiourea, 20 mM Tris base, pH 8.5) and pistils. Lysed samples were subsequently incubated five times in a sonication bath for 30 s with a resting period on ice (30 s). Afterward, lysates were centrifuged for 15 min at 15.000 *g* at 4°C, and the resulting supernatant was transferred to a new reaction tube. Protein concentration was determined via Bradford‐assay. Proteins were subsequently digested into peptides as described in Oertzen‐Hagemann et al. (2019). In brief, 20 µg of lysate were reduced and alkylated in digestion buffer containing 50 mM ammonium bicarbonate. Reduction and alkylation of cysteines was achieved by the addition of 15 mM DTT and sample incubation at 60°C for 30 min, followed by the addition of 5 mM IAA at RT in the dark for 30 min. Trypsin was added as a digestion enzyme in a ratio of 1:40. Digestion was carried out overnight at 37°C and stopped by acidification. The sample was completely dried in a vacuum centrifuge and dissolved in 0.1% TFA. Peptide concentration was determined by amino acid analysis (May et al. 2021), and 200 ng of peptides were used for subsequent mass spectrometric measurements.

### 2.5 Mass Spectrometric Measurements

Peptides of 200 ng were injected using a Vanquish Neo UHPLC system (Thermo Fisher Scientific) with a PepMap Neo C18 Trap Cartridge (300 µm × 0.5 cm, particle size 5 µm) and separated on an analytical column (DNV PepMap Neo, 75 µm × 150 mm, C18, particle size 2 µm, pore size 100 Å). Peptides were separated with a flow rate of 400 nL/min and a solvent gradient from 1% B to 21% B (B: 84% acetonitrile, 0.1% FA) for 70 min, with a subsequent increase up to 40% for 25 min and washing for 5 min with 95% B. Peptides were ionized by ESI and injected into an Orbitrap Fusion Lumos mass spectrometer (Thermo Fisher Scientific). The measurement was carried out by data‐independent acquisition (DIA). Full MS spectra were acquired in the range of 350 to 1400 *m/z* with a resolution of 120,000 (normalized AGC target 250%, maximum injection time 50 ms, RF lens 45%). MS/MS fragments were generated by HCD, with a fixed NCE of 32. MS/MS fragments were acquired in the range from 400 to 900 *m/z* at a resolution of 30,000 in an Orbitrap analyzer (normalized AGC target 1000%, 54 ms maximum injection time). For DIA, a window size of 15 *m/z*, with a 1 *m/z* overlap, was chosen, resulting in 32 windows. The mass spectrometry proteomics data have been deposited to the ProteomeXchange Consortium (Deutsch et al. 2023) via the PRIDE partner repository (Perez‐Riverol et al. 2022) with the dataset identifier PXD055206.

### 2.6 Data Analysis

Mass spectrometry data analysis was carried out in Spectronaut (version 16.5.221115.53000, Biognosys). The directDIA option was chosen and BGS settings were chosen for data analysis, with the exception that trypsin was added as digestion enzyme, carbamidomethylation of cysteines was set as fixed modification due to sample preparation and oxidation of methionine was set as variable modification. Resulting labelfree quantified (LFQ) data were extracted from Spectronaut, and statistical analysis was carried out in Perseus. LFQ data were log2 transformed to achieve a normal distribution of LFQ values and stringent filter criteria were applied, whereby only proteins present in at least three replicates per each group were used for relative quantification. Remaining missing values were replaced from the normal distribution, resulting in a df value of nine for every protein. To determine significantly regulated proteins between the two groups (CTRL, CORT), unpaired Student's *t*‐test was applied, whereby proteins with a *p‐*value <0.05 were considered statistically significant. To identify proteins being of higher or lower abundance, the ratio between LFQ values of each protein was calculated between the groups (CORT/CTRL), resulting in a fold change for each protein (see Table S2). Gene ontology (GO) analysis was carried out in DAVID (Huang et al. 2009; Sherman et al. 2022) as described in Eggers et al. (2021). As a significance threshold, only terms with a *p*‐value <0.05 were considered. Additionally, protein count and fold enrichment were used for interpretation (see Table S3).

We further used a Python script run in Google Colab on Python 3.10 to identify potential GC response elements in genes that we suspected to be directly regulated by the GR. The script utilizes Biopython (Cock et al. 2009) and scanned 5000 bp upstream and downstream of the transcription start site (TSS). Regions within 1000 bp upstream of the TSS and 100 bp downstream of the TSS were defined as the promoter region. A positive GC response element was defined as AGAACA(N0‐3)TGTTCT (Schiller et al. 2014), and a negative response element was defined as CTCC(N0‐2)GGAGA (Surjit et al. 2011).

### 2.7 Quantitative RT‐PCR

RNA isolation was performed using the NucleoSpin RNA/Protein Kit (Macherey & Nagel), according to its handbook. To perform transcriptomic analyses by qPCR, the isolated RNA was transcribed into complementary DNA (cDNA). This was completed according to the manufacturer's protocol using GoScriptTM Reverse Transcriptase and random primers (Promega). Total RNA of 1000 ng was loaded for each cDNA reaction in a working volume of 20 µL. Final cDNA samples were diluted 1:10 with nuclease‐free water and stored at −20°C prior to further processing. To further validate the mass spectrometry data, the quantification of gene expression was performed using quantitative real‐time PCR (qRT‐PCR). The target‐specific primer pairs (see Table S1) were ordered from ThermoFisher Scientific. Lyophilized primers were diluted to 100 mM stock solutions and stored at −20°C.

GoTaq qPCR Master Mix (Promega) and target‐specific primers were used to evaluate the relative mRNA expression levels of myelin basic protein (MBP) and 2′,3′‐cyclic‐nucleotide 3′‐phosphodiesterase [CNP] with the QuantStudio 3 Real‐Time‐PCR system (Thermo Fisher Scientific). qPCR runs were performed at 95°C for denaturation, 60°C for annealing/extension, and 40 cycles as described in Ceylan et al. (2021). With the dye‐based qRT‐PCR, the fluorescence of specific dyes (SYBR Green) in the double‐stranded DNA product was measured. The intensity of fluorescence increases proportionally to the amount of template in each PCR cycle. The relative target expression was analyzed by using the Pfaffl method (Pfaffl 2001) and was normalized to actin B (ACTB) and succinate dehydrogenase complex, subunit A (SDHA) as reference genes. In addition, the relative expression of target genes was expressed as fold change compared to the control group (CTRL). All samples were performed in duplicate, and the mean *Ct* value was used for the calculation. Statistical analysis was performed using Welch's *t*‐test for the comparison of two groups with GraphPad Prism 10 (GraphPad Software Inc.).

### 2.8 Statistical Methods

Normality of the qPCR data was assessed using the Shapiro–Wilk test, which indicated no significant deviation from a normal distribution. Therefore, Welch's *t*‐test was applied for group comparisons. Given the limited reliability of normality tests in small sample sizes, we additionally performed Mann–Whitney *U* tests as a nonparametric alternative to confirm the robustness of the results. For GC fluorescence, five standardized ROIs (50 × 50 px) were placed in anatomically comparable areas across both control and stress conditions using ImageJ. Mean gray values extracted from the green channel confirmed a marked reduction of GR signal in the stress group (mean ± SD: 12.8 ± 1.1) compared to controls (31.6 ± 2.0). Shapiro–Wilk tests were used to assess normality, and Welch's *t*‐tests were conducted.

The statistical analysis of qPCRs, GR intensity, and area measurements was conducted in GraphPad Prism 10 (GraphPad Software Inc.). All statistical analyses, as well as raw data can be found in Table S4.

## 3 Results

To study the influence of prenatal stress, chicken embryos (day 14 of development) were used,‐ whereby prenatal stress was mimicked by injecting corticosterone at day 6 of embryonic development (CORT). In a first step, we aimed to broadly study the morphology of CTRL and CORT‐stressed brains and secondly determine molecular alterations caused by corticosterone treatment.

### 3.1 Investigation on the Morphological Impact of Prenatal Corticosterone Exposure Reveals Alteration in Brain Size

**FIGURE 1:**
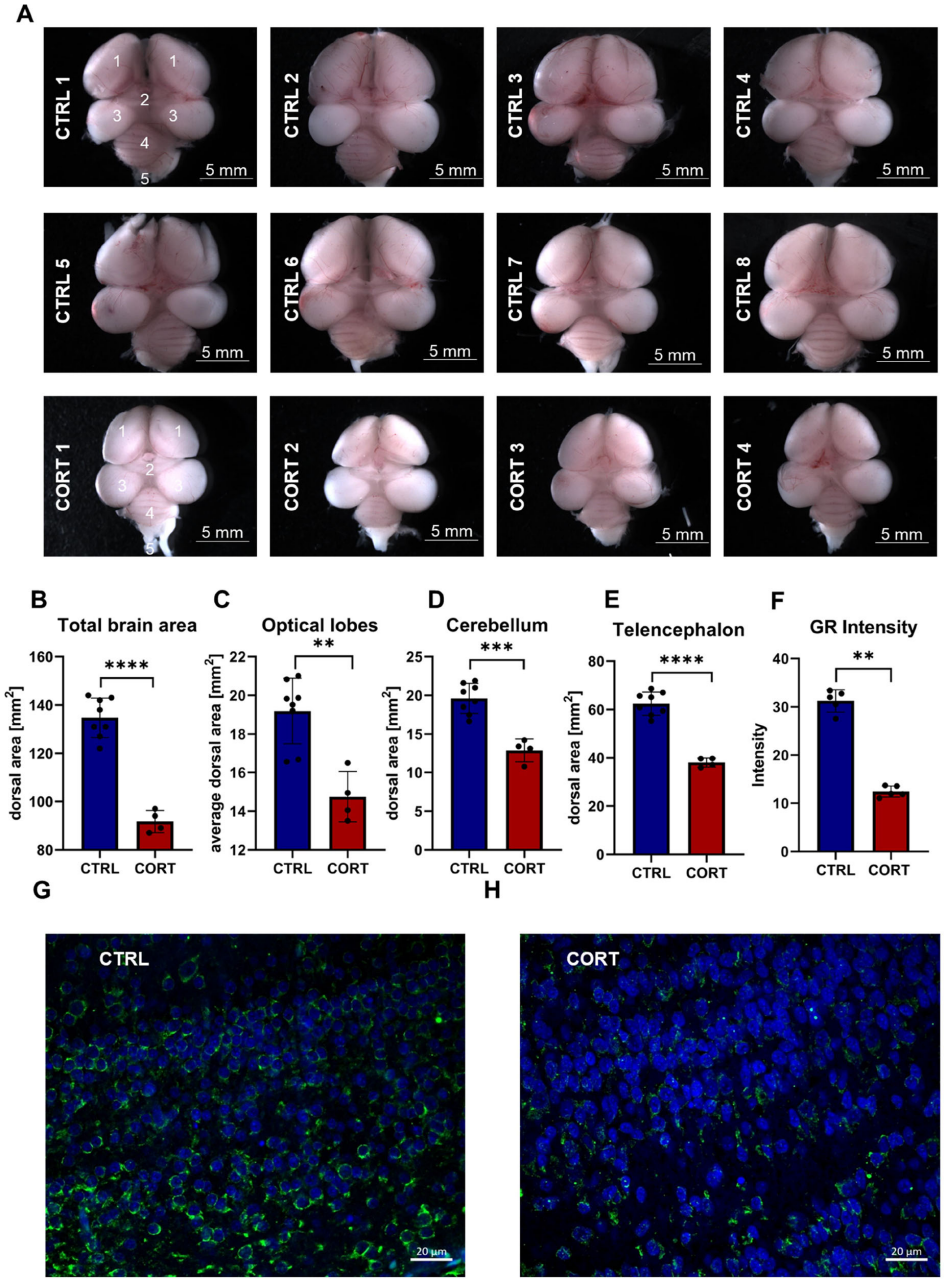
Morphological assessment of control (CTRL) and corticosterone (CORT)‐exposed embryonic chicken brains. (A) Microscopic images of CTRL (*n* = 8, in blue) and CORT‐exposed (*n* = 4, in red) embryonic chicken brains (Telencephalon (1), Pineal gland (2), Optic lobes (3), Cerebellum (4), Medulla oblongata (5)). (B–E) Size measurements of the respective brain areas B. Total brain area (*p‐*value: 0.0001; *t*: 12.49, df: 9.8444; *U*‐value: 0) (C) average optical lobes (*p*‐value: 0.002; *U*‐value: 5), (D) cerebellum (*p*‐value: 0.004; *U*‐value: 0), (E) telencephalon (*p* value: 0.004; *U*‐value: 0). All brain areas were found to be significantly reduced in the CORT‐stressed group (**p‐value <0.05, Welch's *t*‐test). Photos have been taken with a 0.79× magnification. Part (F) depicts the results of the quantitative analysis of GR immunofluorescence (*n* = 5, *p*‐value: <0.0001, *t*: 16.26, df: 5.837, Welch's *t*‐test). Part (G) shows the GR expression in a frontal section of the midbrain of control embryos, where the GR signal is prominently distributed throughout the neural tissue, indicating a robust presence of the receptor under normal conditions. Part (H) presents the GR expression in the midbrain of embryos treated with corticosterone. In comparison to the control (F), there is a noticeable reduction in GR signal intensity, suggesting a downregulation or decreased availability of the receptor following corticosterone treatment. Raw data and statistics can be found in Table S4.

Isolated brains of CTRL (*n* = 8) and CORT‐stressed chicken embryos (*n* = 4) were first assessed on the level of morphology (see Figure 1). As brains were extracted at day 14 of development, a visible segmentation of the forebrain into telencephalon (1), pineal gland (2), optic lobes (3), cerebellum (4), and medulla oblongata (5) was present in CTRL animals (see Figure 1A). In CORT‐stressed animals, however, the telencephalic lobes appeared more compact (see Figure 1A). Further, a significant reduction in area was observed in the CORT group for all brain areas, clearly pointing towards a link between corticosterone exposure and delays in brain development (see Figure 1B–E, **p‐value <0.02). Additionally, we investigated the abundance of GR in CTRL and CORT‐stressed midbrains on the level of IHC (see Figure 1F–G), whereby in CTRL animals, the GR signal is found to be prominently distributed throughout the neural tissue, indicating a robust presence of the receptor under normal conditions (see Figure 1G). In comparison to the control, a noticeable reduction in GR signal intensity in CORT‐stressed animals can be observed (see Figure 1H), suggesting a downregulation or decreased availability of the receptor following corticosterone treatment. To assess potential region‐specific alterations in GR expression in a quantitative manner, we conducted a semiquantitative analysis of IHC intensity in midbrain sections (see Figure 1F,G). Using ImageJ, five standardized ROIs (50 × 50 px) were placed in anatomically comparable areas across both control and stress conditions. Mean gray values extracted from the green channel confirmed a marked reduction of GR signal in the stress group (mean ± SD: 12.8 ± 1.1) compared to controls (31.6 ± 2.0).

### 3.2 Molecular Alterations Caused by Prenatal Corticosterone Exposure Highlight Deficits in Oligodendrocyte Development and Myelination

As the present study belongs to the first ones to investigate the proteomic composition of the developing chicken brain, we aimed in a first step to elucidate the comparability in protein quantification of the current study (6580 identified proteins, see Table S2) and a pioneering study led by Stepler et al. reporting 5929 proteins. Protein overlap analysis (based on gene names) revealed a substantial overlap of over 50% (3952 proteins) between the two studies, whereby 1553 proteins were uniquely identified by Stepler et al. and 2137 proteins were solely quantified in the present study. The overlap of 50% is remarkable, especially in regard to the fact that chicken brains utilized in the two studies were isolated at different stages of development; hence, overlapping proteins may eventually be considered the stable proteome of the developing brain. To elucidate differences in the brain proteomes assessed, we conducted a GO enrichment (GO‐term) analysis based on cellular compartments (CC) on proteins identified in both studies and study‐specific proteins (see Table S3). Commonly identified proteins could be annotated to a plethora of terms essential for brain architecture and cell function. GO terms comprising the highest number of associated proteins were found to be the cytoplasm (1047 proteins), the nucleus (906 proteins), the cytosol (615 proteins), the mitochondrion (295 proteins), and the endoplasmic reticulum (165 proteins), supporting the idea that common proteins may depict the stable proteome of the developing brain.

Proteins solely identified in the dataset of Stepler et al. were found to be enriched in terms associated with the nucleus and various protein complexes. Among them are complexes essential for RNA‐protein interactions (MSL complex, fold enrichment (13.4)), DNA mismatch repair (MutLalpha complex, fold enrichment (13.4)), microtubule organization (HAUS complex, fold enrichment (11.2)), and nucleus architecture (NSL complex, fold enrichment (9.6)), mirroring the early developmental status of the chicken brain analyzed in their study. In contrast, terms found to be enriched in the current study were associated, among others, with proteins essential for the synapse. Of the Top25 enriched terms, 18 terms were associated with synaptic compartments and processes, such as synaptic cleft (fold enrichment 7.3), GABA‐ergic synapse (fold enrichment 6.0), the postsynapse (fold enrichment 5.2), and the myelin sheath (fold enrichment 4.6), highlighting a more mature developmental stage (see Table S3).

**FIGURE 2:**
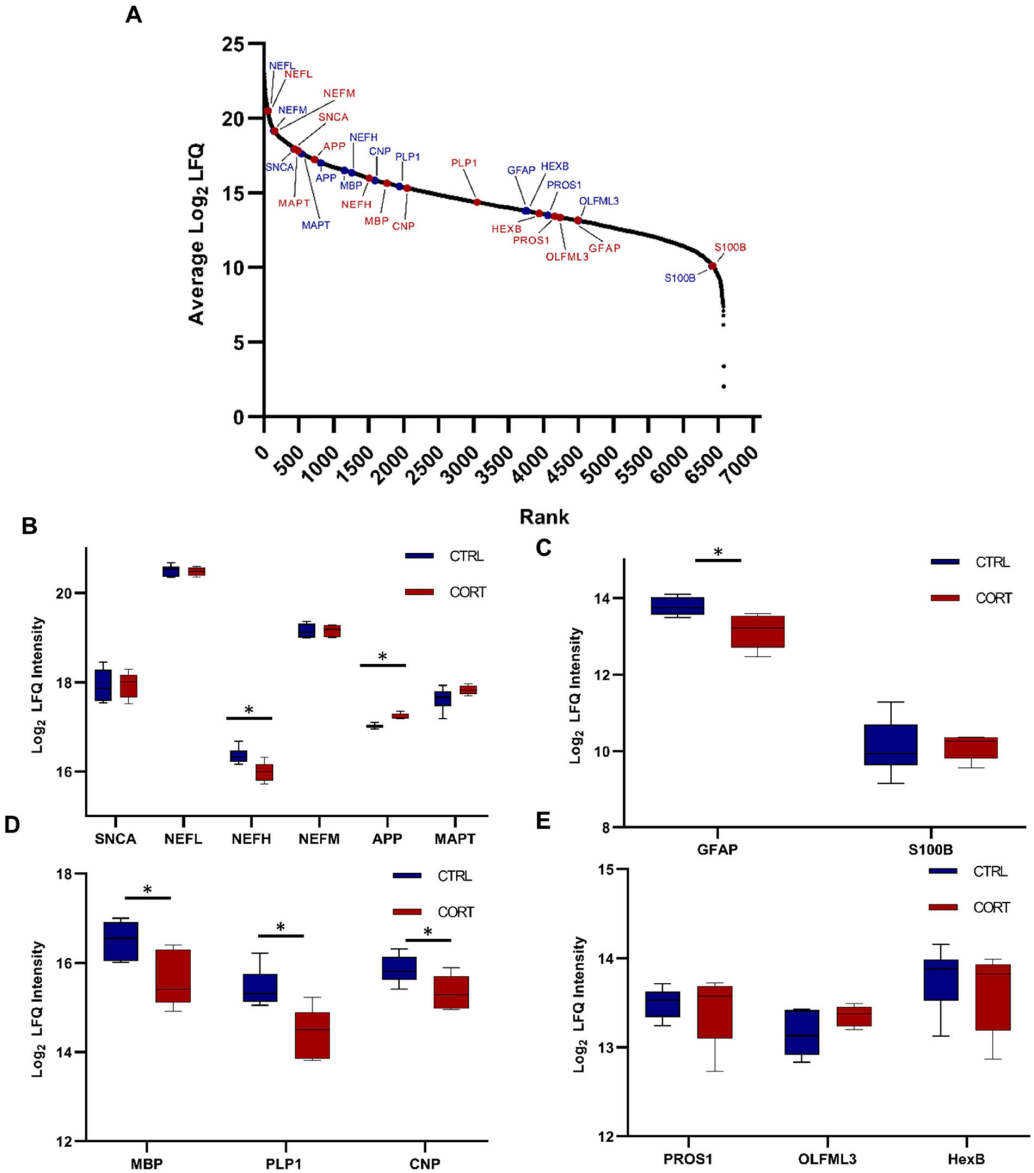
**Alteration in the expression of marker proteins for the different cell types present within the brain**. (A) Ranking plot showcasing the position of cell type marker proteins for control (CTRL) samples in blue and corticosterone‐stressed samples (CORT) in red. Especially the oligodendrocytic markers MBP, CNP, and PLP1, as well as the astrocytic marker GFAP, display a shift in rank in the CORT group. (B) Boxplots displaying the log2 label‐free quantified (LFQ) values of cell type marker proteins, based on mass spectrometric measurements (B: neuronal marker, C: astrocytic marker, D: oligodendrocytic marker, E: embryonic microglia marker). Proteins being significantly differentially expressed are marked with an asterisk (Student's *t*‐test *p*‐value <0.05). Raw data and statistics can be found in Table S4.

In a second step, we aimed to investigate whether corticosterone exposure may influence the abundance of cell types within the brain. To do so, we determined the abundance of well‐known marker proteins of cell types of the brain (see Figure 2), among them neuronal (alpha‐synuclein (SNCA), neurofilament light and medium chain (NEFL, NEFM), amyloid precursor protein (APP), microtubule‐associated protein tau (MAPT), see Figure 2A,B), astrocytic (glial fibrillary acidic protein (GFAP), protein S100B (S100B), see Figure 2A,C), oligodendrocytic (MBP, myelin proteolipid protein (PLP1), CNP, see Figure 2A,D) and embryonic microglia‐markers (beta‐hexosaminidase subunit beta (HEXB), olfactomedin‐like protein 3 (OLFML3), protein S (PROS1), see Figure 2A, E).

To determine the abundance level of marker proteins, rank plots of all quantified proteins were created, ranking proteins in dependence of their averaged LFQ values. Subsequently, the rank of marker proteins was visualized for each group (see Figure 2A (in blue: CORT, in red: CTRL)), and boxplots (based on LFQ values) were used to showcase the abundance levels of highlighted proteins for all analyzed samples within one group. With this, we were able to gain a first insight into the abundance of cell types. The expression values of the neuronal markers SNCA, NEFL, NEFM, and MAPT seemed to be comparable for both groups (see Figure 2B). The distribution of abundance values within the groups was highly similar, with the exception of SNCA, for which a greater variation within a group was observed. The neuronal markers NEFH and APP instead were found to be significantly differential between the groups, whereby an increased expression of APP (*p*‐value: 0.0002) and a significant reduction of NEFH in the CORT‐treated group were observed (*p*value: 0.01). For astrocytic markers instead, a significant difference in abundance could be visualized for GFAP (*p*‐value: 0.01), but not for S100B (see Figure 2C). Interestingly, all three investigated oligodendrocytic markers displayed a significantly higher abundance in CTRL brains compared to the CORT‐stressed group (MBP *p*‐value: 0.02, PLP1 *p*value: 0.007, CNP *p*‐value: 0.03, see Figure 2D). The investigated embryonic microglial markers all displayed a high variation within the same group, with no clear differences in abundance between CTRL and CORT (see Figure 2E).

**FIGURE 3:**
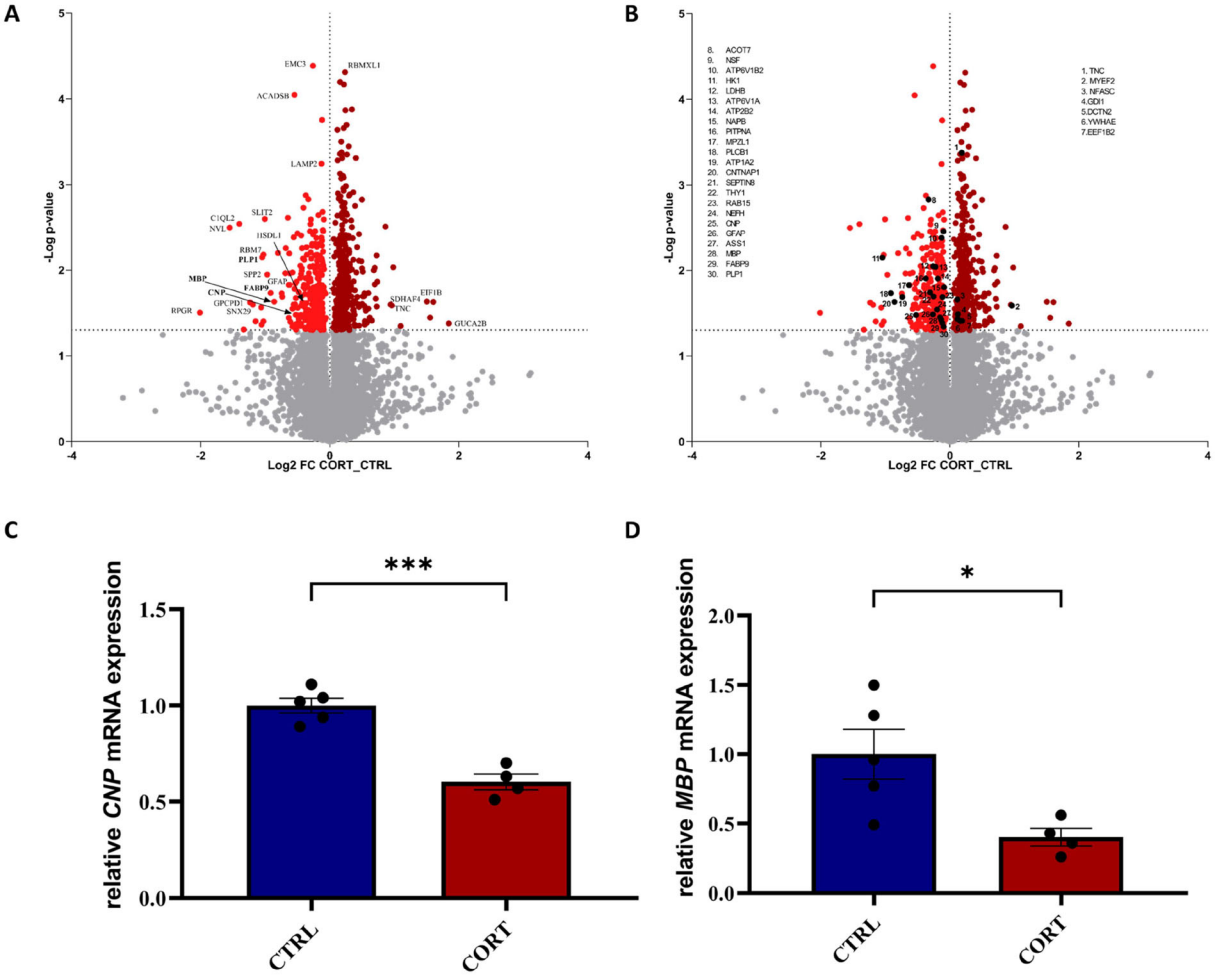
Differential protein abundance and qPCR validation. On the *x*‐axis the fold change is displayed as log2 ratio between label‐free quantified (LFQ) values of corticosterone‐exposed (CORT) and control (CTRL) samples. On the *y*‐axis, the −log *p* value is displayed and the significance threshold is indicated by the dotted line. (A) Proteins being of higher abundance in the CORT group are displayed in dark red, and proteins being of lower abundance in the CORT group in light red. The Top20 proteins are numbered and associated via gene name. (B) Significantly regulated myelin‐associated proteins are marked in black. Significantly regulated proteins are numbered and associated via gene name. (C and D) qPCR displaying the relative mRNA expression of CNP (C, *p*‐value: 0.0002159, *t*: 7.087, df: 6.737, *U*‐value, 0) and MBP (D, *p*‐value: 0.02259317, *t*: 3.144, df: 4.949, *U‐*value: 1) (C, *p*‐value: 0.0159, *U*‐value: 0) and MBP (D, *p*‐value: 0.0317, *U*‐value: 1) in CTRL (blue) and CORT (red)‐stressed brain. For both mRNAs, a significant decrease in CORT animals was observed (*p‐value <0.05).

In a final step, we aimed to compare the proteomes of CTRL and CORT‐stressed animals by the means of a quantitative comparison to determine proteins being significantly changed in their abundance. Of 6580 proteins, 643 were found to be significantly differentially expressed, whereby 360 were found to be of increased abundance in CORT‐stressed brains and 283 of lower abundance compared to the CTRL (see Figure 3A). To gain an overview, we investigated the Top20 proteins with the highest fold changes first (see Tables 1 and 2). Proteins found to be of higher abundance in CORT‐stressed brains could be mainly annotated to proteins associated with transcription regulation and remodeling of chromatin. Interestingly, two acute phase proteins, hemopexin and tenascin C (TNC), were found to be increased by over threefold, alongside two embryonic hemoglobins, which are assigned as acute phase proteins as well, potentially indicating neuroinflammation.

**TABLE 1:** Proteins identified as being of higher abundance in corticosterone (CORT, *n* = 5)‐exposed brains compared to an unstressed control (CTRL, *n* = 6).

Protein name	Gene name	*p*‐value	FC CORT/CTRL	Function (UniProt)
Guanylate cyclase activator 2B	GUCA2B	0.042	3.591	Natriuretic function
Hemopexin	n.a.	0.024	3.043	Acute phase response
Fibrillin 2	n.a.	0.036	2.939	Calcium‐binding microfibril
Eukaryotic translation initiation factor 1B	EIF1B	0.023	2.836	Translation factor
Single‐stranded DNA binding protein 4	SSBP4	0.045	2.142	DNA replication, repair
SECIS binding protein 2 like	SECISBP2L	0.009	1.977	Regulation of selenoprotein expression
Tenascin C	TNC	0.026	1.945	Acute phase response, glycoprotein, extracellular matrix
Succinate dehydrogenase assembly factor 4	SDHAF4	0.025	1.923	Respiratory chain complex II
Pygopus family PHD finger 1	PYGO1	0.003	1.817	Wnt pathway
Small ribosomal subunit protein uS10m	MRPS10	0.014	1.665	Ribosome
Orthodenticle homeobox 2	OTX2	0.027	1.655	Transcription factor
Bromodomain containing 3	BRD3	0.007	1.652	Remodeling of chromatin structures
Hemoglobin subunit pi	n.a.	0.022	1.638	Early embryonic hemoglobin
RNA binding motif (RNP1, RRM) protein 3	RBM3	0.009	1.621	RNA binding
Sp3 transcription factor	SP3	0.015	1.621	Transcription factor
RUNX1 translocation partner 1	RUNX1T1	0.010	1.607	Transcription factor, immunomodulatory
Solute carrier family 38 member 1	SLC38A1	0.039	1.574	Ion exchanger
Chromatin complexes subunit BAP18	CUNH17	0.036	1.534	Chromatin complex component
E3 ubiquitin‐protein ligase listerin	LTN1	0.028	1.515	Ribosome quality control complex
Hemoglobin subunit epsilon	HBE	0.019	1.515	Early mammalian embryonic hemoglobin

*Note*: Fold changes (FC) were calculated by the ratio of label‐free quantified values (LFQ) of CORT divided by CTRL samples. Significance was determined by unpaired Student's *t*‐test, whereby a *p‐*value <0.05 was considered statistically significant. Information on protein name and gene name is given, as well as on protein function (based on UniProt entry).

**TABLE 2:** Proteins identified as being of lower abundance in corticosterone (CORT, *n* = 5)‐exposed brains compared to an unstressed control (CTRL, *n* = 6).

Protein name	Gene name	*p*‐value	FC CORT/CTRL	Function (UniProt)
Retinitis pigmentosa GTPase regulator	RPGR	0.031	−4.038	Photoreceptor integrity
Nuclear VCP‐like	NVL	0.003	−2.930	Pre‐rRNA processing
Complement C1q like 2	C1QL2	0.003	−2.644	Acute phase response
Ribonuclease homolog	n.a.	0.049	−2.523	Unknown
Glycerophosphocholine phosphodiesterase 1	GPCPD1	0.024	−2.353	Phospholipid biosynthesis
Sorting nexin 29	SNX29	0.025	−2.284	Protein transport
C‐type lectin domain‐containing protein	LOC121113327	0.040	−2.220	Involved in the routing of antigen for presentation to T cells
Protein‐tyrosine sulfotransferase	TPST2	0.027	−2.090	*O*‐sulfation of tyrosine residues
Large ribosomal subunit protein uL16m	MRPL16	0.043	−2.079	Ribosome
Proteolipid protein 1	PLP1	0.007	−2.067	Major myelin protein CNS
RNA binding motif protein 7	RBM7	0.007	−2.039	RNA binding
Beta‐galactosidase	GLB1L2	0.040	−2.039	Glycosyl transfer
Slit guidance ligand 2	SLIT2	0.003	−2.010	Axon guidance
Secreted phosphoprotein 24	SPP2	0.011	−1.959	Bone remodeling
Myelin P2 protein	FABP9	0.019	−1.888	Lipid transport protein in Schwann cells
Myelin basic protein	MBP	0.023	−1.816	Components of the myelin membrane in CNS
Protein arginine methyltransferase 9	PRMT9	0.006	−1.743	Alternative splicing of pre‐mRNA
Peroxisomal biogenesis factor 11 beta	PEX11B	0.019	−1.677	Peroxisomal proliferation
Argininosuccinate synthase	ASS1	0.020	−1.669	Biosynthesis of arginine
Epiplakin 1	LOC121109863	0.011	−1.612	Cytoskeletal linker

*Note*: Fold changes (FC) were calculated by the ratio of label‐free quantified values (LFQ) of CORT divided by CTRL samples. Significance was determined by unpaired Student's *t*‐test, whereby a *p‐*value <0.05 was considered statistically significant. Information on protein name and gene name is given, as well as on protein function (based on UniProt entry).

In concordance with our cell type analysis of the Top20 proteins being of lower abundance in CORT‐stressed brains, three proteins were found to be associated with the myelin sheath, namely, PLP1, myelin P2 protein (FABP9), and MBP. Additionally, a phosphodiesterase essential for phospholipid synthesis was found to be decreased. Other Top20 proteins were associated with neuronal maintenance, such as protein transport and axon guidance, as well as RNA processing and binding.

As several myelin‐associated proteins were found to be decreased in CORT‐exposed embryos, we aimed to comprehensively study the expression profile of myelin‐associated proteins within our dataset. For that, we oriented ourselves on a publication aiming to investigate the molecular anatomy of the insulating myelin sheath (Jahn et al. 2009) on the level of proteins. Jahn et al. identified 294 myelin‐associated proteins, of which 30 were found to be differential in our dataset (see Figure 2, 3 and Table S2). Of those 30 proteins, 23 were found to be decreased in CORT‐stressed brains,‐ and seven were found to be increased (see Figure 3B). Next to the already observed myelin‐associated proteins in the Top20 analysis, CNP (FC: 1.4) was found to be decreased. CNP is a membrane‐anchored myelin enzyme and is particularly expressed in the CNS, playing a major role in myelination (Raasakka and Kursula 2014). A closer examination of upregulated myelin‐associated proteins revealed that two proteins, namely, TNC (FC: 1.9) and myelin expression factor 2 (MYEF2, FC: 1.1), are both thought to have inhibitory effects on myelin production, either via transcriptional repression of MBP or via inhibition of oligodendrocyte differentiation, potentially indicating alterations in oligodendrocyte maturation under corticosterone exposure. However, we need to stress that reported fold changes were minor, not exceeding two‐fold for all differential myelin‐associated proteins. Thus, we aimed to verify the observed effect on myelin‐associated proteins at the level of mRNA and IHC. Confirmatively, quantitative PCR of CNP and MBP showed a significant decrease (*p*‐value: <0.05) of relative mRNA expression in CORT‐exposed brains, solidifying our proteomic observations (see Figure 3C,D). Further staining of MBP suggested a diminished staining in CORT‐stressed brains (see Figure S1 and S2).

### 3.3 Alterations in Transcription Factors and Signaling Proteins Associated With the GR and Glia Cell Differentiation

As our molecular characterization revealed a profound effect of corticosterone‐induced stress on glia cells, in particular oligodendrocytes, we aimed to shed light on potential molecular mechanisms causing developmental delays in oligodendrocyte maturation and thus myelination. For that, we investigated all significantly expressed proteins in respect to their connection to glia cell maturation. Indeed, major MAPK signaling proteins were found to be significantly regulated, however, with only mild fold changes (see Table 3). Among them are tyrosine‐protein kinase FYN, as well as the mitogen‐activated protein kinase 1 (MAP2K1), the stress‐activated protein kinase JNK, and the kinase enhancer actin filament‐associated protein 1‐like 2 (AFAP1L2). Furthermore, several serine/arginine‐rich splicing factors (SRp) that are known to influence GR mRNA translation and are heavily involved in the regulation of its isoforms via alternative splicing were found to be significantly downregulated in the CORT group. Among these SRp's were SRp40 (SRSF5), SRp20 (SRSF3), and SRp30a (SRSF1). The protein FKBP51, which is involved in retaining the GR in the cytoplasm, was also found to be significantly downregulated. Notably, hydroxysteroid dehydrogenase 1 like (HSDL1) was found to be upregulated in the CORT group.

**TABLE 3:** Proteins identified as being differentially expressed between corticosterone (CORT, *n* = 5)‐exposed brains and unstressed controls (CTRL, *n* = 6), associated with the GR and glia cell differentiation.

Protein name	Gene name	*p*‐value	FC CORT/CTRL
Actin filament‐associated protein 1 like 2	AFAP1L2	0.036	1.1
Eukaryotic translation initiation factor 4E	EIF4E	0.033	1.1
Hydroxysteroid dehydrogenase like 1	HSDL1	0.024	−1.3
Mitogen‐activated protein kinase kinase 1	MAP2K1	0.034	−1.1
Peptidylprolyl isomerase	FKBP51	0.006	−1.2
Serine and arginine rich splicing factor 3	SRSF3	0.028	1.1
Serine and arginine rich splicing factor 4	SRSF4	0.001	1.2
Serine and arginine rich splicing factor 5	SRSF5	0.006	1.2
Splicing factor, arginine/serine‐rich 1	SRSF1	0.001	1.2
Stress‐activated protein kinase JNK	MAPK10	0.032	1.1
Tyrosine‐protein kinase	FYN	0.031	−1.1

*Note*: Fold changes (FC) were calculated by the ratio of label‐free quantified values (LFQ) of CORT divided by CTRL samples. Significance was determined by unpaired Student's *t*‐test, and a *p*‐value <0.05 was considered statistically significant. Information on protein name and gene name is given, as well as on protein function (based on UniProt entry).

## 4 Discussion

Behavioral studies have robustly demonstrated that maternal stress during pregnancy leads to both short‐ and long‐term adverse effects on offspring, particularly in neurological development, indicating that prenatal stress may profoundly influence brain development (Maccari et al. 2003; Talge et al. 2007; Weinstock 2008). So far, the exact molecular mechanisms leading to neurological alterations are not completely understood. Thus, in this study, we aimed to elucidate molecular factors contributing to developmental delays and neurological alterations in an avian model organism of prenatal stress. Our model was specifically designed to simulate an acute prenatal stressor through a single corticosterone exposure at a defined developmental stage. This approach enabled a clear temporal association between the exposure and subsequent neurodevelopmental outcomes, minimizing confounding factors such as prolonged systemic alterations or developmental retardation.

Using our mass spectrometry‐based approach, we have gained valuable insights into the mid‐to‐late developmental stages of the chicken embryo, representing the most comprehensive cerebral proteomic analysis of this organism to date, with 6580 proteins identified. The results of this study confirm the deleterious effects of early prenatal corticosterone exposure that have been proposed in earlier studies. Macroscopic analysis of the prepared embryos revealed significant reductions in brain size across all investigated regions in corticosterone‐exposed chicken embryos. However, due to major differences in brain size, we chose to analyze whole brains to determine said reduction, rather than brain sections, not taking the curvature of the brains into consideration. Nonetheless, the observed reduction may be attributed to delayed myelination, as myelin is highly abundant in the mature brain, comprising more than 25% of its total weight. To support this finding, staining of MBP was carried out. Unfortunately, the majority of commercially available antibodies are not validated for use in chicken tissue, and therefore, specific stainings may lead to suboptimal results. Nevertheless, we were able to confirm a reduction in myelin‐positive regions in corticosterone‐exposed brains on the level of IHC (see Figures S1 and S2). Similar alterations in hippocampal myelination have also been observed in rat and guinea pig models exposed to prenatal stress (Crombie et al. 2023; Xu et al. 2013). In addition, in comparison to the control, a noticeable reduction in GR signal intensity in CORT‐stressed animals was observed, suggesting a downregulation or decreased availability of the receptor following corticosterone treatment. However, this observation could not be corroborated on the level of mass spectrometry, because, at most, only one peptide corresponding to the GR could be identified, hindering accurate quantification. This is likely a result of technical limitations in DIA mass spectrometry, where low‐abundant peptides tend to remain unidentified. A second limitation to be noticed is that we exclusively focused on GR expression. Given the important complementary role of mineralocorticoid receptors (MRs) in regulating corticosterone effects (McCann et al. 2021), future studies should investigate both receptor systems to provide a more comprehensive understanding of prenatal stress‐induced alterations in brain development.

### 4.1 Cell‐Specific Impacts

On the molecular level, corticosterone was found to have an impact on the development of glial cells. In the central nervous system, four major types of glial cells can be found: oligodendrocytes, astrocytes, microglia, and ependymal cells. Although no markers were found to be regulated for the latter two, a strong downregulation of the major oligodendrocyte marker proteins, MBP, CNP, and PLP1, was observed in the corticosterone‐exposed group. Similarly, studies utilizing prolonged corticosterone treatment to induce stress in adult rats highlighted a suppression in oligodendrocyte precursor cell proliferation (Alonso 2000). Other studies reported GC‐mediated modulations of oligodendrocyte maturation during brain development (Kumar et al. 1989). Thus, our analyses revealed a marked downregulation of oligodendrocyte markers; however, this finding could reflect several potential underlying processes, including general delayed brain development, a specific delay in oligodendrocyte or glial cell maturation, or even an inhibition of glial cell development. Further investigation is needed to differentiate between these possibilities. Supporting the hypothesis of corticosterone's inhibitory effect on overall glial cell differentiation is the observation that GFAP, an astrocyte marker, was significantly reduced in corticosterone‐exposed brains (FC: −1.6), whereas neuroepithelial stem cell protein (Nestin), a marker associated with neural progenitor cells, was upregulated (FC: 1.3). The upregulation of glial maturation factor (GMFB, FC: 1.2) (Zaheer et al. 1993) in corticosterone‐exposed embryos suggests that, at the time of investigation, glial progenitor cells may be in the process of differentiating into mature glial cells. This observation supports the hypothesis that corticosterone exposure may cause a delay in, rather than a complete inhibition of, glial cell differentiation. Myelination in the chicken embryo begins around embryonic day 10 (E10), with notable progress by E12 when compact myelin starts to form. This process accelerates significantly between E12 and E15, as the first oligodendrocytes begin myelinating and take on a Schwann cell‐like appearance (Anderson et al. 2000). Given this timeline, E14 represents a critical phase in myelination, making it an optimal time point to investigate the effects of corticosterone exposure on this process. Although neither the untreated nor the treated brains displayed complete myelination at E14, this stage is ideal for capturing key molecular events during myelination. Nevertheless, additional time‐dependent molecular studies, investigation of later developmental stages, and cell culture‐based functional studies in primary oligodendrocytes are essential to further validate and expand upon the findings, particularly as most observed fold changes were modest, not exceeding two‐fold for the majority of regulated proteins. Additionally, global phospho‐proteomic approaches are inevitable to assess activation or deactivation of enzymes present in relevant signaling pathways (Wilson et al. 2018).

We further need to acknowledge that prenatal stress *in vivo* often manifests as a more chronic condition. Chronic exposure paradigms, potentially involving repeated corticosterone administrations over extended developmental periods, could provide important complementary insights into how sustained stress affects brain development and myelination. Such models would allow for the investigation of cumulative effects but also present challenges, such as difficulties in temporally isolating specific developmental events and the potential emergence of compensatory or retardation phenomena. Moreover, it is important to note that both acute and chronic stress involve complex physiological responses beyond elevations in GC levels. Activation of the autonomic nervous system, inflammatory pathways, and other neuroendocrine changes contribute significantly to the overall stress response. Thus, although corticosterone exposure captures an essential component of prenatal stress, it represents only one aspect of the broader biological processes involved. Future studies employing chronic exposure models and more integrative approaches will be relevant to fully elucidate the complex nature of prenatal stress effects on neurodevelopment.

**FIGURE 4:**
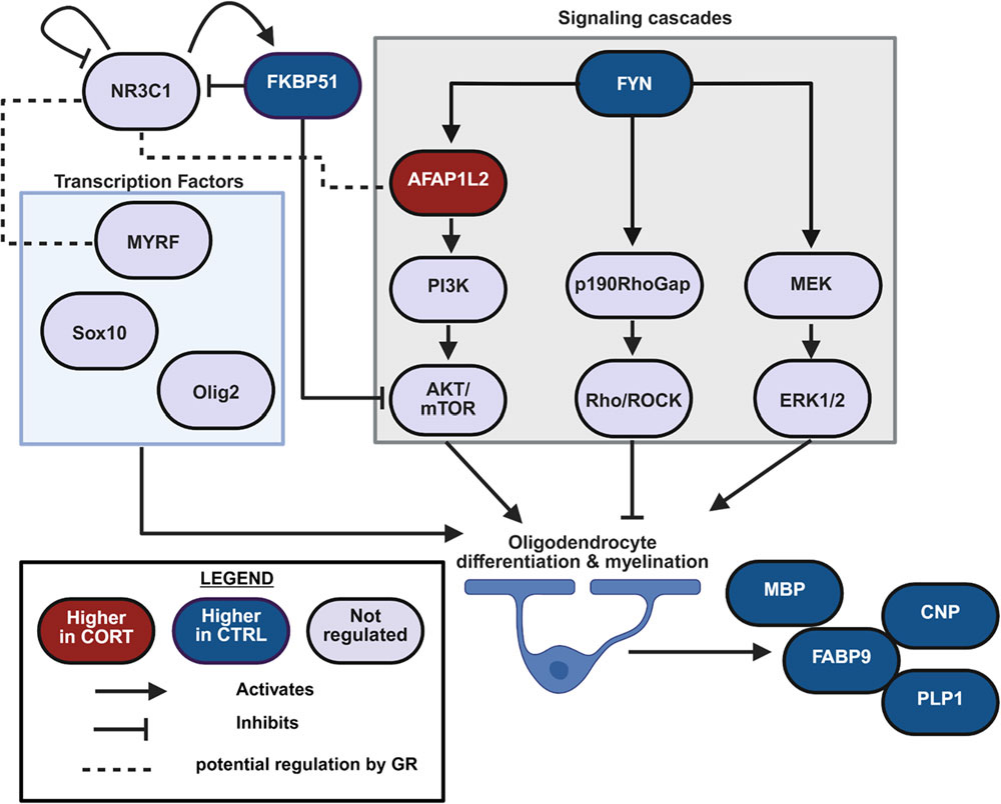
Proposed mechanism of corticosterone‐induced impairment of oligodendrocyte maturation and myelination. Proteins in red were more abundant in CORT‐exposed embryos; proteins in blue were more abundant in CTRL embryos; purple indicates proteins not significantly altered or undetected. The figure highlights possible GR‐mediated regulation of FYN kinase, AKT/mTOR, and MEK/ERK signaling, and compensatory responses via AFAP1L2 and FKBP51. *Source*: Graphic created with BioRender.com.

In spite of that, with our comprehensive proteomic characterization of the stressed embryonic brain, we aimed to formulate a potential mechanism for the GC‐mediated inhibition of glial cell differentiation (see Figure 4). Nonetheless, we must emphasize that the observed fold changes were modest — none exceeded 1.5‐fold — and therefore our hypothesis should be validated through further investigation.

### 4.2 Potential Mechanism of GC‐Mediated Inhibition of Glial Cell Differentiation

Previous studies have shown that GC administration can lead to increased expression of glial markers such as MBP and PLP and promote morphological changes consistent with cellular maturation in the hippocampus of adult rats (Chetty et al. 2014). In line with our study, paradoxically, administration of high GC concentrations in a developmental context seems to reduce myelination, oligodendrocyte maturation, and proliferation (Zia et al. 2015).

A study by Tanga et al. (2019) suggested a potential pathway that may influence oligodendrocyte maturation as well as myelination. Their investigation proposes that oligodendrocyte differentiation is heavily influenced by the RhoA/ROCK pathway, the PI3K/AKT/mTOR pathway, as well as the MEK/ERK pathway.

The main regulator of these pathways is the tyrosine kinase FYN. It activates OPC differentiation and myelination by regulating all of the aforementioned pathways. In addition to initiating differentiation, FYN is instrumental in modulating the translation of key myelin proteins (Tanga et al. 2019). FYN‐deficient mice exhibit deficits in oligodendrocyte maturation, reduced numbers of mature myelinating cells, and diminished expression of myelin proteins such as MBP, leading to hypomyelination in the brain and underlining the deep involvement of FYN in oligodendrocyte maturation (Goto et al. 2008). A recent dissertation showed that GC treatment led to a repression of FYN across all tissues in mice (Ourailidou 2017). In‐line with this, we have observed a mild decrease in abundance of this crucial protein, delivering a possible explanation for the reduced expression of myelin‐associated proteins identified in this study. In silico, we identified three potential negative GC response elements within the gallic FYN gene, indicating that FYN may be directly regulated by the GR (Genomic positions: 6538181, 65392209, and 65449915 in ENSGALG00015012028).

In our analysis, we found AFAP1L2 to be mildly upregulated upon GC stress. Knockdowns of this protein are known to induce apoptosis in OPCs (Tanga et al. 2019). Furthermore, AFAP1L2 is known to activate the PI3K/AKT/mTOR pathway by binding to a regulatory subunit of PI3K (Tanga et al. 2019). An increased abundance of AFAP1L2 and subsequently higher activity of the PI3K/AKT/mTOR phosphorylation cascade could therefore be a plausible rescue mechanism to counteract the decreased abundance of the major oligodendrocyte‐associated proteins MBP, CNP, FABP9, and PLP1. We identified a potential GC response element (CTCCGGAGA) in the promoter region of the AFAP1L2 gene (ENSGALG00010022436) in silico, 882 bp from the TTS, indicating a direct regulation of AFAP1L2 by the GR. This observation should, however, in the future be verified using Electrophoretic mobility shift assays or other suitable methods.

Additionally, we could demonstrate a mild downregulation of FKBP51 upon GC stress. The downregulation of FKBP51 upon GC exposure is typically not observed and represents a discrepancy between this study and the existing literature. However, this observation may be explained by a rebound effect, as other studies investigating FKBP51 expression after GC exposure typically examine the expression after only a couple of hours of treatment, or by a partial loss of GR sensitivity after high GC exposure. FKBP51 is an essential protein that blocks the GC binding site of the GR in the cytosol and is also directly regulated by the GR (Mendonça et al. 2023). FKBP51 is further known to be an important negative regulator of the AKT/mTOR pathway by acting as a scaffolding protein that facilitates the recruitment of the phosphatase PHLPP to AKT, thus leading to dephosphorylation of AKT and thereby downregulating AKT signaling (Wang 2011). A GC‐induced downregulation of FKBP51 may lead to increased signal transduction in the AKT/mTOR pathway and thus activate oligodendrocyte differentiation, resulting in increased expression of myelin‐associated proteins such as MBP, CNP, PLP1, and FABP9. Both FKBP51 downregulation and AFAP1L2 upregulation may represent compensatory efforts to restore oligodendrocyte maturation during periods of heightened GC stress. Nevertheless, these responses might be of limited efficacy, as the AKT/mTOR pathway is modulated by numerous other factors, and oligodendrocyte maturation and myelination are also governed by additional signaling pathways and transcriptional regulators. As already stated above, studies investigating acute and chronic stress models will be of highest interest to clarify whether observed alterations in proteins are a direct result of acute corticosterone exposure or rather rescue mechanisms trying to counteract the loss of myelination and oligodendrocyte‐associated proteins. Additionally, phospho‐proteomic studies will be able to shed light on dynamic changes in signal transduction. Furthermore, key oligodendrocytic transcription factors — including Sox10, Olig2, and MYRF — were not detected in our analysis. Consequently, we are unable to draw conclusions regarding their regulatory responses to GC exposure. However, in silico, we identified potential GC response elements in intronic regions of MYRF (ENSGALG00015014781), indicating the possibility of a direct regulation of this transcription factor.

Previous studies in guinea pigs have indeed shown that this oligodendrocyte deregulation persists into early childhood but recovers by mid to late childhood (Bennett et al. 2017). Nevertheless, reduction of mature oligodendrocytes in early childhood and during critical phases of development could permanently affect brain composition and behavior despite recovery. Our findings suggest that FYN downregulation after prenatal corticosterone stress may lead to a misregulation of the AKT/mTOR pathway, the Rho/ROCK pathway,‐ as well as the MEK/ERK pathway and subsequent impaired maturation of oligodendrocytes.

## 5 Conclusion

This study provides important insights into how corticosterone‐mediated prenatal stress influences avian neurodevelopment. These findings may have broader implications for understanding human brain development under comparable stress conditions. By conducting one of the most comprehensive proteomic analyses to date, we identified significant deficits in myelination as a key pathological hallmark associated with prenatal stress. These findings align with observations in mammalian models, reinforcing the critical role of GC signaling in neural development. Our results suggest that the timing of corticosterone exposure is crucial, as it coincides with critical phases of myelination, particularly around embryonic day 14. Although the observed molecular changes were modest, they underscore the importance of further time‐dependent studies to fully understand the impact of prenatal stress on neurodevelopment. Future studies should investigate the impact of chronic stress, extend these findings to later developmental stages, and explore functional outcomes. Such work will deepen our understanding of how early‐life stressors shape neurological trajectories.

## Author Contributions

**Morris Gellisch**: conceptualization, project administration, chicken egg incubation, corticosterone exposure and isolation of brain, data curation, immunohistochemistry, data analysis (images), writing – original draft. **Britta Eggers**: conceptualization, project administration, mass spectrometric measurements, data analysis, data curation, data analysis (images), writing – original draft. **Beate Brand‐Saberi**: project administration, supply of laboratory equipment. **Katrin Marcus**: project administration, supply of laboratory equipment. **Simon Faissner**: supply of laboratory equipment, (q)PCR, immunohistochemistry. **Gabriela Morosan‐Puopolo**: chicken egg incubation,. corticosterone exposure and isolation of brain, immunohistochemistry. **Bela Gaertner**: data analysis, (q)PCR, immunohistochemistry, data analysis (images), writing – original draft. **Charmaine Schücke**: (q)PCR, immunohistochemistry. **Katharina Klöster**: data analysis, immunohistochemistry. **Darius Saberi**: data analysis (images).

## Conflicts of Interest

The authors declare no conflicts of interest.

## Supporting information



Supplementary Table: dneu23003‐sup‐0001‐TableS1_.xlsx

Supplementary Table: dneu23003‐sup‐0002‐TableS2.xlsx

Supplementary Table: dneu23003‐sup‐0003‐TableS3.xlsx

Supplementary Table: dneu23003‐sup‐0004‐Table 4.xlsx

Selection of MBP and DAPI staining on brain cryosections. The sections contain the optic lobe with the myelin‐rich stratum opticum (1) and the stratum album centrale (3). Furthermore, the cerebellum (2) and the Fasciculus longitudinalis medialis (4), a prominent nerve fiber tract in the midbrain. A negative control (D) was conducted on a control brain section in order to test unspecific binding of the secondary antibody. No distinct secondary antibody staining could be observed here. Artifacts are marked with an asterisk (*). Distinct antibody binding can be observed in all other sections. When comparing the MBP staining of CORT sections (A and C) to CTRL sections (B), it becomes evident that MBP expression is reduced, mainly in the optic lobes of CORT brains. Artifacts were removed with Canva AI. The original figure can be found as Figure S2.

## Data Availability

The data have been deposited to the ProteomeXchange Consortium (https://proteomecentral.proteomexchange.org/ui) via the PRIDE partner repository (https://www.ebi.ac.uk/pride/) with the dataset identifier PXD055206.
