# Supplementary material for: Neurodevelopmental Impact of Prenatal Stress: A Proteomic Analysis of Myelination Disruptions in the Avian Embryo
Source: Dev Neurobiol. 2025 Oct 14;85(4):e23003. doi: 10.1002/dneu.23003 (PMC12521880; doi:10.1002/dneu.23003)
Supplement: Supplementary file 5 — Selection of MBP and DAPI staining on brain cryosections. The sections contain the optic lobe with the myelin‐rich stratum opticum (1) and the stratum album centrale (3). Furthermore, the cerebellum (2) and the Fasciculus longitudinalis medialis (4), a prominent nerve fiber tract in the midbrain. A negative control (D) was conducted on a control brain section in order to test unspecific binding of the secondary antibody. No distinct secondary antibody staining could be observed here. Artifacts are marked with an asterisk (*). Distinct antibody binding can be observed in all other sections. When comparing the MBP staining of CORT sections (A and C) to CTRL sections (B), it becomes evident that MBP expression is reduced, mainly in the optic lobes of CORT brains. Artifacts were removed with Canva AI. The original figure can be found as Figure S2. [file DNEU-85-0-s001.pdf]

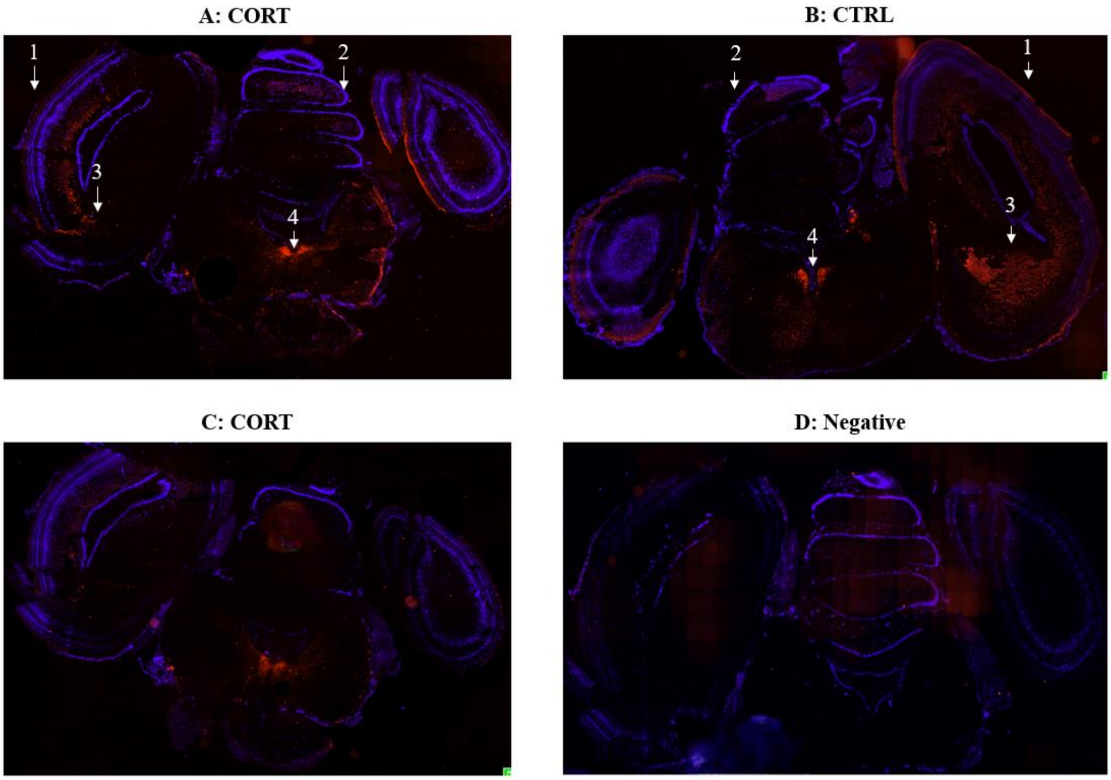

Supplementary figure S1: Selection of MBP, and DAPI staining on brain cryosections. The sections contain the optic lobe with the myelin rich *stratium opticum* (1), and the stratum *album centrale* (3). Furthermore, the *Cerebellum* (2), and the *Fascius longitudinalis medialis* (4), a prominent nerve fiber tract in the midbrain. A negative control (D) was conducted on a control brain section in order to test unspecific binding of the secondary antibody. No distinct secondary antibody staining could be observed here. Artifacts are marked with an asterisk (*). Distinct antibody binding can be observed in all other sections. When comparing the MBP staining of CORT sections (A and C) to CTRL sections (B) it becomes evident that MBP expression is reduced, mainly in the optic lobes of CORT brains. Artifacts were removed with Canva AI. The original figure can be found as supplementary figure S2.

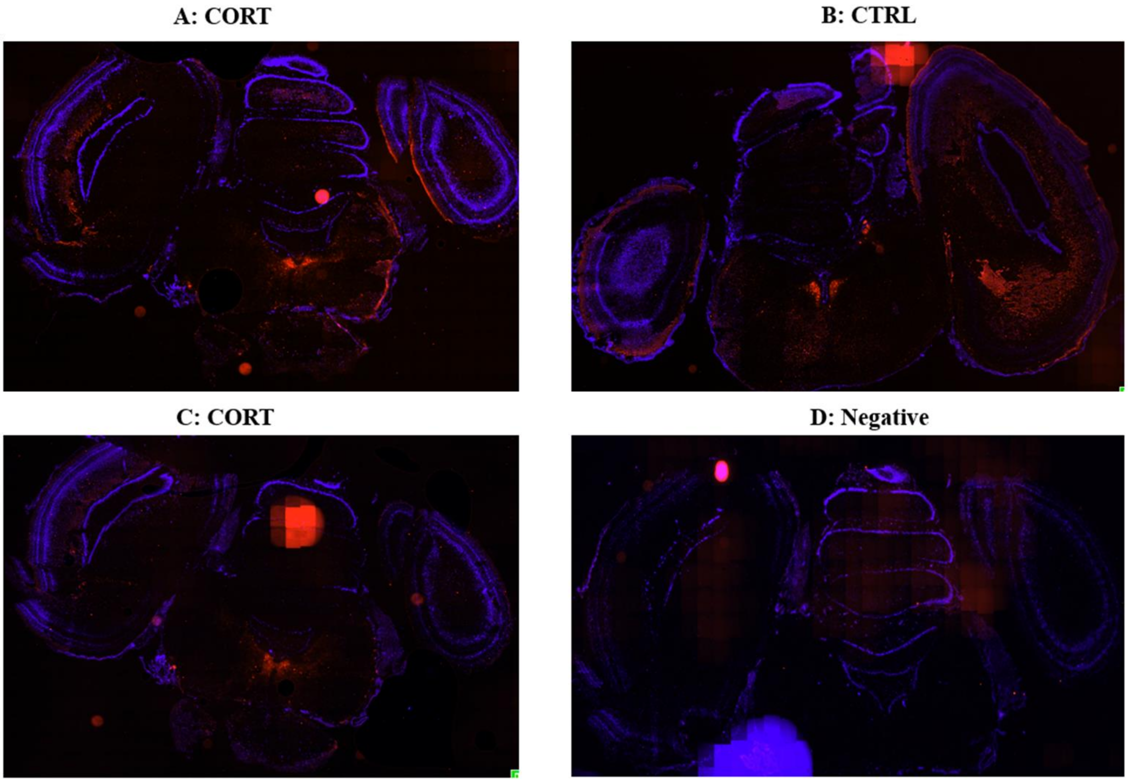

Supplementary figure S2: Original figure
